# Development and Validation of a Nomogram Model for Predicting the Risk of Readmission in Patients with Heart Failure with Reduced Ejection Fraction within 1 Year

**DOI:** 10.1155/2022/4143173

**Published:** 2022-09-16

**Authors:** Yue Hu, Xiaotong Wang, Shengjue Xiao, Chunyan Huan, Huimin Wu, Tao Xu, Minjia Guo, Hong Zhu, Defeng Pan

**Affiliations:** ^1^Department of General Practice, The Affiliated Hospital of Xuzhou Medical University, Xuzhou, Jiangsu 221004, China; ^2^Department of Cardiology, The Affiliated Hospital of Xuzhou Medical University, Xuzhou, Jiangsu 221004, China; ^3^Department of Cardiology, Zhongda Hospital, School of Medicine, Southeast University, 87 Dingjiaqiao, Nanjing, Jiangsu 210009, China

## Abstract

The high incidence of readmission for patients with reduced ejection fraction heart failure (HFrEF) can seriously affect the prognosis. In this study, we aimed to build a simple predictive model to predict the risk of heart failure (HF) readmission in patients with HFrEF within one year of discharge from the hospital. This retrospective study enrolled patients with HFrEF evaluated in the Heart Failure Center of the Affiliated Hospital of Xuzhou Medical University from January 2018 to December 2020. The patients were allocated into the readmission or nonreadmission group, according to whether HF readmission occurred within 1 year of hospital discharge. Subsequently, all patients were randomly divided into training and validation sets in a 7 : 3 ratio. A nomogram was established according to the results of univariate and multivariate logistic regression analysis. Finally, the area under the receiver operating characteristic curve (AUC-ROC), calibration plot, and decision curve analysis (DCA) were used to validate the nomogram. Independent risk factors for HF readmission of patients with HFrEF within 1 year of hospital discharge were as follows: age, body mass index, systolic blood pressure, diabetes mellitus, left ventricular ejection fraction, and angiotensin receptor-neprilysin inhibitors. The AUC-ROC of the training and validation sets were 0.833 (95% confidence interval (CI): 0.793-0.866) and 0.794 (95% CI: 0.727-0.852), respectively, which have an excellent distinguishing ability. The predicted and observed values of the calibration curve also showed good consistency. DCA also confirmed that the nomogram had good clinical value. In conclusion, we constructed an accurate and straightforward nomogram model for predicting the 1-year HF readmission risk in patients with HFrEF. This nomogram can guide early clinical intervention and improve patient prognosis.

## 1. Introduction

Heart failure (HF) is the ultimate outcome of various heart diseases, which seriously affect people's quality of life [1]. According to epidemiologic studies, HF has become an epidemic disease, with more than 38 million patients with HF worldwide [2]. With the aging of the population, the prevalence of HF increases, exceeding 10% in people over 70 years of age [3, 4]. HF has high mortality and low survival rates comparable to malignant tumors, and the 5-year survival rates for men and women with HF were 25% and 38%, respectively [5].

The 2021 European Society of Cardiology (ESC) Guidelines for the diagnosis and treatment of acute and chronic HF classified HF based on the left ventricular ejection fraction (LVEF) as follows: heart failure with reduced ejection fraction (HFrEF) (LVEF ≤ 40%), heart failure with mildly reduced ejection fraction (HFmrEF) (LVEF = 41–49%), and heart failure with preserved ejection fraction (HFpEF) (LVEF ≥ 50%) [6]. HFrEF, also known as systolic HF, accounts for about 50% of patients with HF [7]. A study has shown that compared with HFmrEF and HFpEF, HFrEF has a higher mortality rate and worse prognosis [8]. HF is the main reason for admission and readmission in patients over 65 years old, and the readmission rate within one year for HF exacerbations is as high as 35% [9–11]. Studies have shown that the decline in physical status in patients with HF is closely related to repeated readmissions and not only leads to the decline of cardiac function but also affects the patient's treatment compliance, which creates an enormous economic burden for patients and the healthcare system [12]. Therefore, accurate recognition of patients' risk of readmission within 1 year and early intervention is critical to patient outcomes.

A nomogram is a visualized model which can transform complex regression equations into visual graphs, and it is widely used for disease diagnosis and prognosis [13]. In predicting readmission or survival rate in patients with HF, studies have shown that the nomogram is an ideal model that can reduce readmission and mortality [14, 15]. However, there have been no studies of HF readmission in patients with HFrEF. Therefore, we wanted to construct a nomogram to predict the 1-year risk of HF readmission in HFrEF, which could guide clinical diagnosis, advance intervention, and improve patients' quality of life.

## 2. Methods

### 2.1. Study Population and Design

This retrospective study was based on the database of the Heart Failure Center, The Affiliated Hospital of Xuzhou Medical University. Patients diagnosed with HFrEF according to the 2016 ESC Guidelines from January 2018 to December 2020 were enrolled [16]. This study was conducted according to the principles of the Declaration of Helsinki and approved by the Medical Research Ethics Committee of the Affiliated Hospital of Xuzhou Medical University (approval number XYFY2022-KL094-01). Because the study was a single-center retrospective study, the review committee waived the requirement for written informed consent.

The inclusion criteria were as follows: (1) HFrEF diagnosed according to the 2016 ESC Guidelines for the Diagnosis and Treatment of Acute and Chronic HF [16] and (2) New York Heart Association (NYHA) classification of cardiac function levels II to IV. The exclusion criteria were as follows: (1) patients lost to follow-up for various reasons, (2) patients with missing critical clinical data, (3) a history of malignant tumor, (4) patients with severe end-stage disease of essential organs such as the liver, kidney, or brain, and (5) other reasons. The Heart Failure Center has established a follow-up system for all patients, and all follow-up data can be accessed. The study's endpoint was defined as HF readmission within 1 year of hospital discharge.

### 2.2. Predictor Variables

Through a review of the literature, we collected various factors that may influence patient prognosis, including demographic data, comorbidities, hematologic indicators, echocardiographic indicators, medication at admission, and device therapy. As shown in Table 1, a total of 43 parameters were obtained at admission. All indicators were obtained within 24 hours of admission.

### 2.3. Statistical Analysis

In this study, R version 3.6.4, Stata version 13.0, and SPSS version 22.0 were used for statistical analysis. The measurement data conforming to the normal distribution were expressed as mean ± standard deviation (*X* ± *S*), and the independent sample *t*-test was used for intergroup comparison. Non-normally distributed data were represented by the median (*M*) and interquartile ranges (*M* (P25, P75)), and nonparametric tests were used for intergroup comparison. For the assessment of normality, we used the Shapiro-Wilk test. The counting data were expressed as frequency and percentage (%), and the chi-square test was used for intergroup comparison. *P* < 0.05 indicates statistical significance. A nomogram was established according to the results of univariate and multivariate logistic regression analysis. The area under the receiver-operating characteristic (AUC-ROC) curve was used to verify the discrimination of the nomogram, and a bootstrap self-sampling method (*B* = 1000) was used to internally validate the model and plot calibration curves. Finally, decision curve analysis (DCA) was used to confirm the clinical benefit of this nomogram.

## 3. Results

### 3.1. Baseline Characteristics

**Figure 1:**
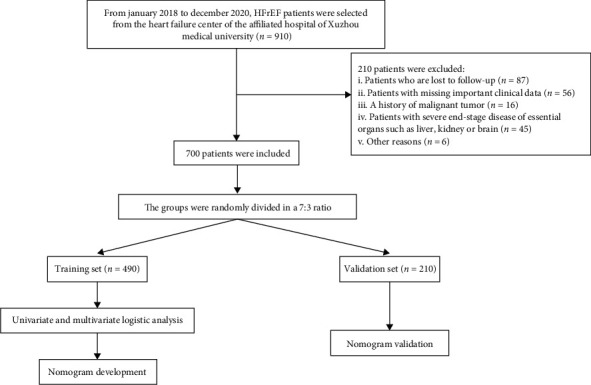
Flow chart of inclusion and exclusion process of HFrEF patients.

From January 2018 to December 2020, there were 910 patients with HFrEF in the Heart Failure Center of Affiliated Hospital of Xuzhou Medical University. Based on the inclusion and exclusion criteria, 700 patients were eventually enrolled. The patients were divided into readmission and nonreadmission groups according to whether they were readmitted for HF within 1 year. Moreover, we randomly divided all patients into training (*n* = 490) and validation (*n* = 210) sets in a ratio of 7 : 3. The patient selection process is shown in Figure 1.

The baseline data of these patients are shown in Table 1. A total of 217 patients were readmitted, with an end-point event rate of 31%. The mean age of the patients in the readmission group was 65.0 ± 12.7, of whom 153 (70.5%) were male, compared with 60.0 ± 15.2 and 321 (66.5%) in the nonreadmission group. The variables that showed significant differences between the readmission and nonreadmission groups were as follows: age, NYHA class, body mass index (BMI), systolic blood pressure (SBP), diabetes mellitus (DM), coronary heart disease, anemia, estimated glomerular filtration rate (eGFR), uric acid, NT-proBNP, LVEF, and angiotensin receptor-neprilysin inhibitors (ARNI) (all *P* < 0.05).

### 3.2. Univariate and Multivariate Logistic Analysis of HF Readmission within 1 Year

We included the variables from univariate logistic analysis with *P* < 0.05 in subsequent multivariate logistic analysis (Table 2). Univariate logistic analysis showed that factors associated with HF readmission within 1 year in patients with HFrEF included the following: age, NYHA class, BMI, SBP, DM, coronary heart disease, anemia, eGFR, uric acid, LVEF, and ARNI (all *P* < 0.05). We included these 11 variables into the multivariate logistic analysis, and the results were as follows: age (odds ratio (OR): 1.033; 95% confidence interval (CI): 1.018-1.049), BMI (OR: 0.783; CI: 0.699-0.876), SBP (OR: 0.716; CI: 0.430-1.194), DM (OR: 3.302; CI: 2.182-4.996), LVEF (OR: 0.901; CI: 0.867-0.937), and ARNI (OR: 0.254, CI: 0.172-0.375). These 6 variables were independent risk factors for HF hospital readmission within 1 year in patients with HFrEF.

### 3.3. Clinical Features of the Training and Validation Sets

To prevent overfitting of the clinical predictive model in the analysis of influencing factors, patients with HFrEF were randomly divided into training and validation sets in a ratio of 7 : 3. As shown in Table 3, the training and validation sets were not statistically different in clinical characteristics. This shows that our dataset division is reasonable and comparable.

### 3.4. Development and Validation of the Nomogram

**Figure 2:**
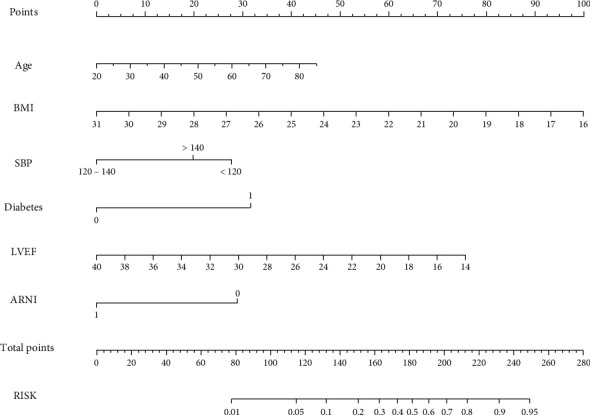
Nomogram used for predicting the risk of readmission in patients with HFrEF within 1 year. BMI: body mass index; SBP: systolic blood pressure; LVEF: left ventricular ejection fraction; ARNI: angiotensin receptor neprilysin inhibitor.

**Figure 3:**
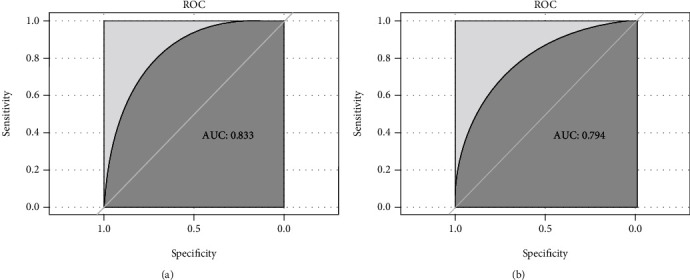
The ROC curves of the clinical predictive model are plotted based on the training set (3A) and validation set (3B). ROC: receiver-operating characteristic; AUC: area under the receiver-operating characteristic.

**Figure 4:**
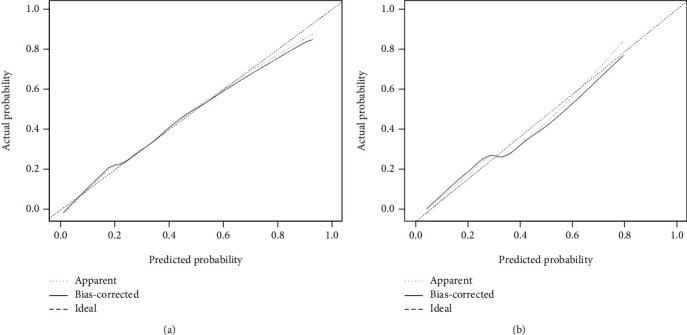
Calibration curve of the nomogram on the data of training set (4A) and validation set (4B).

**Figure 5:**
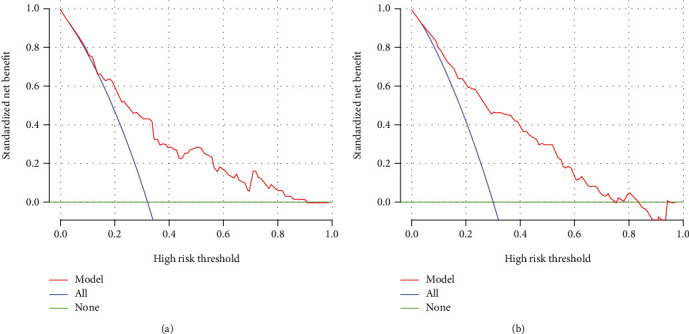
The clinical benefit of the predictive model was evaluated with data from the training set (5A) and the validation set (5B).

Based on the relative weights of the risk factors in Table 2, a nomogram was drawn as shown in Figure 2. For the validation of the nomogram, ROC curves were first drawn for the training and validation set data. The AUC-ROC for the training set was 0.833 (95% CI: 0.793-0.866), and AUC-ROC for the validation set was 0.794 (95% CI: 0.727-0.852). This suggests that the model's discriminative ability was good, as shown in Figure 3. Then, we used the bootstrap self-sampling method to repeat this 1000 times and drew the calibration curves of this nomogram for the training and verification sets. The results showed that the predicted probability of this model is in good agreement with the actual probability and the model calibration is good, as shown in Figure 4. At last, to verify the clinical benefit of the model, DCA curves were drawn for the training and validation set data. According to the DCA curves, the net benefit of the training and verification sets was significantly higher than the two extremes, as shown in Figure 5. Therefore, the nomogram has good clinical benefits.

## 4. Discussion

Studies have shown that patients with HFrEF have a 25.3–35.4% chance of being readmitted to the hospital for HF within 1 year, which is consistent with our study's finding of the 31% HF readmission rate. The high prevalence and mortality of HF have placed a heavy burden on healthcare systems, and the global prevalence of HF is projected to reach 25% by 2030; HFrEF is the type of HF with the worst prognosis [17]. Although medical advances in treating HF have progressed, its prevalence and readmission rates are still increasing [18]. Therefore, early identification of readmission risks for patients with HF and implementation of early intervention is of great significance for patient prognosis [19]. In our study, we found that age, BMI, SBP, diabetes, LVEF, and ARNI were an independent risk factor for HF readmission within 1 year in patients with HFrEF.

The incidence of HF increases with age and is accompanied by changes in the heart structure and function [3, 4, 20, 21]. Ferreira et al. showed that elderly patients with HFrEF had a poor prognosis, and cardiovascular markers positively correlated with age were related to extracellular matrix organization and inflammatory processes [22]. For cardiovascular disease, age is not only a significant risk factor but can even determine the prognosis of HF [23, 24]. Economic development has improved peoples' living standards, and obesity has become a public health concern [25]. A study has shown that a lower BMI is strongly related to an increased risk of all-cause death from cardiovascular disease [26] and low BMI has also been identified as an independent risk factor for all-cause readmission [14].

Hypertension is the most common and important risk factor for HF, and 75% of patients with HF have hypertension, and studies have shown that long-term stable blood pressure control can reduce HF risk by 50% [27, 28]. Studies have shown that both high and low systolic blood pressure will lead to a poor prognosis for patients with HFrEF, which is consistent with our findings [29–31]. DM is common, accounting for about 40% of HF patients and also adversely affects the prognosis of patients with HF [32, 33]. The study by Mac Donald et al. showed that DM was a significant independent predictor of high mortality and HF readmission [34]. The mechanism of how hyperglycemia affects the prognosis of patients with HF has not been fully elucidated; it is speculated to be related to the following factors [35–37]: (1) directly or indirectly affecting myocardial cell function through vascular injury, (2) persistent hyperglycemia-induced oxidative stress leading to cardiomyocyte failure and necrosis, (3) irreversible advanced glycosylation end-products (AGEs), which reduce myocardial contractility and compliance, and (4) diabetic nephropathy may limit the use or uptitration of renin–angiotensin–aldosterone system (RAAS) blockade agents [38].

LVEF is a parameter commonly used to evaluate left ventricular systolic function, which can reliably measure left ventricular function and structure [39]. Studies have shown that LVEF is closely related to the prognosis of patients with HFrEF, and the recovery of ejection fractions can reduce patients' readmission rate and mortality [40, 41], which is consistent with our study. Sacubitril-valsartan is the first dual inhibitor of a novel anti-HF drug called ARNI for treating patients with HFrEF [16]. In this study, ARNI was a protective factor for readmission in patients with HFrEF. Studies have shown that ARNI has not only good efficacy in reversing left ventricular remodeling and reducing hospitalizations associated with HF but also a positive effect on reversing left atrial remodeling [42, 43]. The 2021 ESC Guidelines state that ARNI could further reduce the risk of HF readmission in patients with HFrEF by 21% and the risk of all-cause death by 16% [44].

Our study has some limitations. First, the retrospective cohort design limited this study because of missing important data (8%), participants who were lost to follow up (12%), and missing data on some interesting variables, such as iron status and cystatin-C. In addition, data on ARNI or other medication usage may be underestimated, as these medications may have been initiated after the first HF admission. Those medication changes after discharge and before readmission were not counted because medication use was defined as the medications listed during the first HF admission. Second, this was a single-center study, which lacks external validation.

## 5. Conclusion

We constructed an accurate and simple nomogram for predicting the risk of HF readmission within 1 year in patients with HFrEF. The nomogram can guide early clinical intervention and improve patient prognosis and quality of life. To ensure generality, this model requires external validation.

## Figures and Tables

**Table 1:** Baseline characteristics of the nonreadmission group and readmission group.

Variables	Nonreadmission group (*n* = 483)	Readmission group (*n* = 217)	*P* value
Age (years)	59.95 ± 15.222	64.98 ± 12.646	<0.001
Gender (*n*, %)			0.290
Male	321 (66.5%)	153 (70.5%)	
Female	162 (33.5%)	64 (29.5%)	
NYHA class, (n, %)			0.045
II	81 (16.8%)	22 (10.1%)	
III	321 (66.5%)	149 (68.7%)	
IV	81 (16.8%)	46 (21.2%)	
BMI (kg/m^2^)	21.26 (20.14,22.48)	21.03 (20.10,21.77)	<0.001
SBP (mmHg)			<0.001
<120	104 (21.5%)	91 (41.9%)	
>140	99 (20.5%)	57 (26.3%)	
120–140	280 (58.0%)	69 (31.8%)	
DBP (mmHg)	75 (66,85)	72 (62.5,86)	0.059
>60	327 (67.7%)	131 (60.4%)	
≤60	156 (32.3)	86 (39.6)	
Heart rate (b.p.m.)	74 (63,88)	72 (59,89)	0.196
Smoking (*n*, %)			0.115
No	332 (68.7%)	136 (62.7%)	
Yes	151 (31.3%)	81 (37.3%)	
Drinking (*n*, %)			0.468
No	300 (62.1%)	141 (65%)	
Yes	183 (37.9%)	76 (35%)	
Comorbidities, (*n*, %)			
Hypertension	180 (37.3%)	92 (42.4%)	0.198
Diabetes	94 (19.5%)	91 (41.9%)	<0.001
Atrial fibrillation	60 (12.4%)	39 (18%)	0.051
Stroke	54 (11.2%)	35 (16.1%)	0.069
Coronary heart disease	158 (32.7%)	89 (41%)	0.034
Dyslipidemia	59 (12.2%)	24 (11.1%)	0.662
Myocardial infarction	79 (16.4%)	45 (20.7%)	0.16
COPD	21 (4.3%)	12 (5.5%)	0.495
Chronic kidney disease	15 (3.1%)	9 (4.1%)	0.484
Anemia	150 (31.1%)	84 (38.7%)	0.047
Fasting glucose (mmol/L)	7 (5.45,10.35)	7.11 (5.425,10.33)	0.846
Serum creatinine (*μ*mol/L)	91 (67,137)	90 (71,123)	0.566
eGFR (mL/min)	74.37 (45.24,95.32)	67.84 (47.4,86.79)	0.024
Hemoglobin (g/L)	130 (109,147)	132 (116,150)	0.057
Serum sodium (mmol/L)	139.37 (135.88,142.4)	140 (137.36,142.28)	0.145
Serum kalium (mmol/L)	4.05 (3.67,4.46)	4.1 (3.69,4.515)	0.199
Total cholesterol (mmol/L)	4.19 (3.34,5.52)	4.24 (3.415,5.12)	0.311
LDL-C (mmol/L)	2.56 (1.79,3.49)	2.57 (1.955,3.235)	0.859
Uric acid (*μ*mol/L)	409 (336,537)	438 (342.5, 577.5)	0.048
NT-proBNP (pg/mL)	2338 (1020,5675)	3510 (1452,5868)	0.044
LVEF (%)	31 (28,35)	29 (25.5,33)	<0.001
LVEDd (mm)	72 (65,78)	73 (62,85)	0.134
Medication at admission (*n*, %)			
ACEI	71 (14.7%)	36 (16.6%)	0.52
ARB	110 (22.8%)	36 (16.6%)	0.063
ARNI	329 (68.1%)	83 (38.2%)	<0.001
Beta-blockers	408 (84.5%)	187 (86.2%)	0.559
Aldosterone receptor antagonist	434 (89.9%)	195 (89.9%)	0.998
Ivabradine	1 (0.2%)	2 (0.9%)	0.181
Diuretic	440 (91.1%)	200 (92.2%)	0.64
Digitalis	186 (38.5%)	91 (41.9%)	0.391
Device therapy (*n*, %)			
CRT-D	2 (0.4%)	3 (1.4%)	0.159
CRT-P	2 (0.4%)	2 (0.9%)	0.41
Pacemaker	11 (2.3%)	10 (4.6%)	0.095
ICD	2 (0.4%)	0 (0%)	0.342

BMI: body mass index; SBP: systolic blood pressure; DBP: diastolic blood pressure; COPD: chronic obstructive pulmonary disease; eGFR: estimated glomerular filtration rate; LDL-C: low-density lipoprotein cholesterol; NT-proBNP: N-terminal-pro brain natriuretic peptide; LVEF: left ventricular ejection fraction; LVEDd: left ventricular end-diastolic diameter; ACEI: angiotensin-converting enzyme inhibitor; ARB: angiotensin receptor blockers; ARNI: angiotensin receptor neprilysin inhibitors; CRT-D: CRT defibrillator; CRT-P: CRT pacemaker; ICD: implantable cardioverter defibrillator.

**Table 2:** Univariate and multivariate logistic analysis for the readmission within 1 year.

Variables	Univariate analysis OR (95% CI)	*P* value	Multivariate analysis OR (95% CI)	*P* value
Age (years)	1.025 (1.013,1.037)	<0.001	1.033 (1.018,1.049)	<0.001
NYHA class (*n*, %)		0.048		0.369
II	1.000		1.000	
III	1.709 (1.027,2.845)		1.355 (0.756,2.427)	
IV	2.091 (1.154,3.788)		1.646 (0.824,3.287)	
BMI (kg/m^2^)	0.787 (0.715,0.867)	<0.001	0.783 (0.699,0.876)	<0.001
SBP (mmHg)		<0.001		<0.001
<120	1.000		1.000	
>140	0.615 (0.402,0.942)		0.716 (0.430,1.194)	
120–140	0.225 (0.152,0.333)		0.197 (0.194,0.479)	
Comorbidities (*n*, %)				
Diabetes	2.989 (2.103,4.247)	<0.001	3.302 (2.182,4.996)	<0.001
Coronary heart disease	1.430 (1.028,1.991)	0.034	1.278 (0.856,1.908)	0.230
Anemia	1.402 (1.004,1.959)	0.048	1.487 (0.994,2.223)	0.053
eGFR (mL/min)	0.994 (0.988,0.999)	0.029	1.001 (0.993,1.008)	0.810
Uric acid (*μ*mol/L)	1.001 (1.000,1.002)	0.033	1.001 (1.000,1.002)	0.093
NT-BNP (pg/mL)	1.000 (1.000,1.000)	0.181		
LVEF (%)	0.925 (0.896,0.955)	<0.001	0.901 (0.867,0.937)	<0.001
Medication at admission (*n*, %)				
ARNI	0.290 (0.208,0.405)	<0.001	0.254 (0.172,0.375)	<0.001

BMI: body mass index; SBP: systolic blood pressure; eGFR: estimated glomerular filtration rate; NT-proBNP: N-terminal-pro brain natriuretic peptide; LVEF: left ventricular ejection fraction; ARNI: angiotensin receptor neprilysin inhibitors.

**Table 3:** Baseline characteristics of validation training sets.

Variables	Validation set (*n* = 210)	Training set (*n* = 490)	*P* value
Age (years)	60.39 ± 15.774	61.99 ± 14.13	0.185
Gender (*n*, %)			0.832
Male	141 (67.1%)	333 (68%)	
Female	69 (32.9%)	157 (32%)	
NYHA class (*n*, %)			0.86
II	29 (13.8%)	74 (15.1%)	
III	141 (67.1%)	329 (67.1%)	
IV	40 (19%)	87 (17.8%)	
BMI (kg/m^2^)	21.26 (20.14,22.48)	21.03 (20.10,21.77)	0.449
SBP (mmHg)			0.053
<120	53 (25.2%)	142 (29.0%)	
>140	38 (18.1%)	118 (24.1%)	
120–140	119 (56.7%)	230 (46.9%)	
DBP (mmHg)			0.652
>60	140 (66.7%)	318 (64.9%)	
≤60	70 (33.3%)	172 (35.1%)	
Heart rate (b.p.m.)	73 (62,88.25)	74 (62,88.25)	0.947
Smoking (*n*, %)			0.063
No	151 (71.9%)	317 (64.7%)	
Yes	59 (28.1%)	173 (35.3%)	
Drinking (*n*, %)			0.252
No	139 (66.2%)	302 (61.6%)	
Yes	71 (33.8%)	188 (38.4%)	
Comorbidities (*n*, %)			
Hypertension	70 (33.3%)	202 (41.2%)	0.050
Diabetes	56 (26.7%)	129 (26.3%)	0.925
Atrial fibrillation	27 (12.9%)	72 (14.7%)	0.523
Stroke	34 (16.2%)	55 (11.2%)	0.071
Coronary heart disease	76 (36.2%)	171 (34.9%)	0.743
Dyslipidemia	27 (12.9%)	56 (11.4%)	0.592
Myocardial infarction	37 (17.6%)	87 (17.8%)	0.966
COPD	10 (4.8%)	23 (4.7%)	0.969
Chronic kidney disease	5 (2.4%)	19 (3.9%)	0.319
Anemia	65 (31.0%)	169 (34.5%)	0.363
Fasting glucose (mmol/L)	7.17 (5.3825,10.3275)	7.04 (5.445,10.345)	0.78
Serum creatinine (*μ*mol/L)	92.5 (67.75,132)	89 (68,131)	0.597
eGFR (mL/min)	69.925 (44.2225,92.4325)	72.89 (47.2,92.92)	0.767
Hemoglobin (g/L)	130 (109,147)	132 (116,150)	0.906
Serum sodium (mmol/L)	140 (136,143)	140 (136.6,142.1)	0.841
Serum kalium (mmol/L)	4.04 (3.69,4.4325)	4.075 (3.67,4.48)	0.528
Total cholesterol (mmol/L)	4.28 (3.365,5.3425)	4.2 (3.355,5.315)	0.79
LDL-C (mmol/L)	2.61 (1.8075,3.45)	2.535 (1.825,3.3525)	0.612
Uric acid (*μ*mol/L)	429.5 (346.75,576.25)	413 (332.75,538)	0.084
NT-proBNP (pg/mL)	2433 (953.25,6142.25)	2799 (1180.5,5731.25)	0.524
LVEF (%)	31 (27,34)	31 (27,34)	0.893
LVEDd (mm)	73 (63.75,80)	71 (64,79)	0.628
Medication at admission (*n*, %)			
ACEI	28 (13.3%)	79 (16.1%)	0.168
ARB	49 (23.3%)	97 (19.8%)	0.291
ARNI	128 (61.0%)	284 (58.0%)	0.461
Beta-blockers	185 (88.1%)	410 (83.7%)	0.133
Aldosterone receptor antagonist	187 (89%)	442 (90.2%)	0.642
Ivabradine	1 (0.5%)	2 (0.4%)	0.9
Diuretic	195 (92.9%)	445 (90.8%)	0.377
Digitalis	85 (40.5%)	192 (39.2%)	0.749
Device therapy (*n*, %)			
CRT-D	3 (1.4%)	2 (0.4%)	0.142
CRT-P	1 (0.5%)	3 (0.6%)	0.827
Pacemaker	5 (2.4%)	16 (3.3%)	0.53
ICD	1 (0.5%)	1 (0.2%)	0.537

BMI: body mass index; SBP: systolic blood pressure; DBP: diastolic blood pressure; COPD: chronic obstructive pulmonary disease; eGFR: estimated glomerular filtration rate; LDL-C: low-density lipoprotein cholesterol; NT-proBNP: N-terminal-pro brain natriuretic peptide; LVEF: left ventricular ejection fraction; LVEDd: left ventricular end-diastolic diameter; ACEI: angiotensin-converting enzyme inhibitor; ARB: angiotensin receptor blockers; ARNI: angiotensin receptor neprilysin inhibitors; CRT-D: CRT defibrillator; CRT-P: *CRT* pacemaker; ICD: implantable cardioverter defibrillator.

## Data Availability

The raw data supporting the conclusions of this article will be made available by the authors, without undue reservation.
